# Painful Intra-articular Collagen Fibroma of the Shoulder: A Case Report

**DOI:** 10.7759/cureus.100883

**Published:** 2026-01-05

**Authors:** Kazuhiro Ikeda, Kaishi Ogawa, Shotaro Teruya, Hiromitsu Tsuge, Shinzo Onishi

**Affiliations:** 1 Institute of Medicine, Department of Orthopedic Surgery, University of Tsukuba, Tsukuba, JPN; 2 Department of Orthopedic Surgery, Showa General Hospital, Kodaira, JPN

**Keywords:** collagen fibroma, fibroblastoma, intra-articular tumor, painful tumor, shoulder

## Abstract

Collagen fibroma is a rare benign fibroblastic tumor. It typically arises in the subcutaneous tissue or fascia and is usually asymptomatic; intra-articular occurrence and painful presentation are extremely rare. We report a case of collagen fibroma arising within the shoulder joint and associated with severe pain. A 62-year-old man presented with resting and motion-related pain in the left shoulder. Magnetic resonance imaging performed at another institution demonstrated an intra-articular lesion; however, it was overlooked due to its signal intensity similarity to that of the surrounding subscapularis muscle and joint capsule. When the patient was referred to our institution six years later, the shoulder pain had markedly worsened, with a visual analog scale score of 100 mm. The tumor enlarged between the scapula and the subscapularis muscle, markedly compressing the anterior joint capsule. We performed surgical excision, and histopathological examination confirmed the diagnosis of collagen fibroma. Both resting and motion-related pain resolved rapidly after surgery, and the patient remained recurrence-free during a five-year follow-up period. This case demonstrates that collagen fibroma can arise intra-articularly and cause severe pain, which may lead to diagnostic difficulty. For patients with unexplained shoulder pain, it is essential to perform careful imaging evaluation with consideration of intra-articular tumors.

## Introduction

Collagen fibroma, also known as desmoplastic fibroblastoma, is a rare benign fibrous tumor that was first described by Evans in 1995 [1]. It is classified within the fibroblastic/myofibroblastic tumor group in the latest World Health Organization classification of soft tissue and bone tumors [2]. Collagen fibroma most commonly occurs in the subcutaneous tissue or fascia of middle-aged to older men [3,4,5]. Clinically, collagen fibroma is generally asymptomatic and presents as a slowly growing, painless mass [5,6]; consequently, many patients remain unaware of the lesion for long periods [5]. The tumor is noninfiltrative and does not recur after complete excision, and is therefore regarded as a biologically benign entity [5].

Here, we report an unusual case of collagen fibroma arising within the shoulder joint and associated with severe pain, which remained undiagnosed for approximately six years. This atypical presentation highlights important diagnostic and therapeutic considerations for unexplained shoulder pain.

## Case presentation

**Figure 1:**
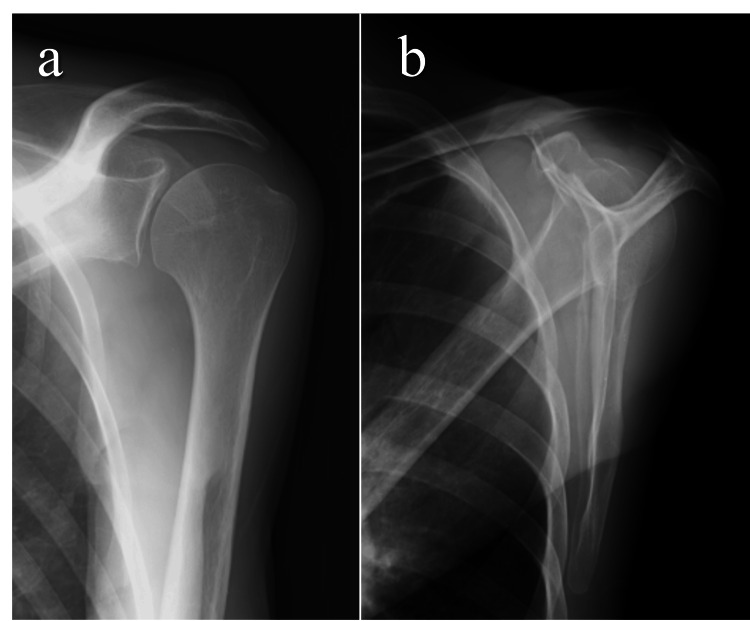
Plain radiographs obtained six years before referral to our institution. (a) Anteroposterior view and (b) scapular Y view showing no apparent osseous abnormalities or joint incongruity.

**Figure 2:**
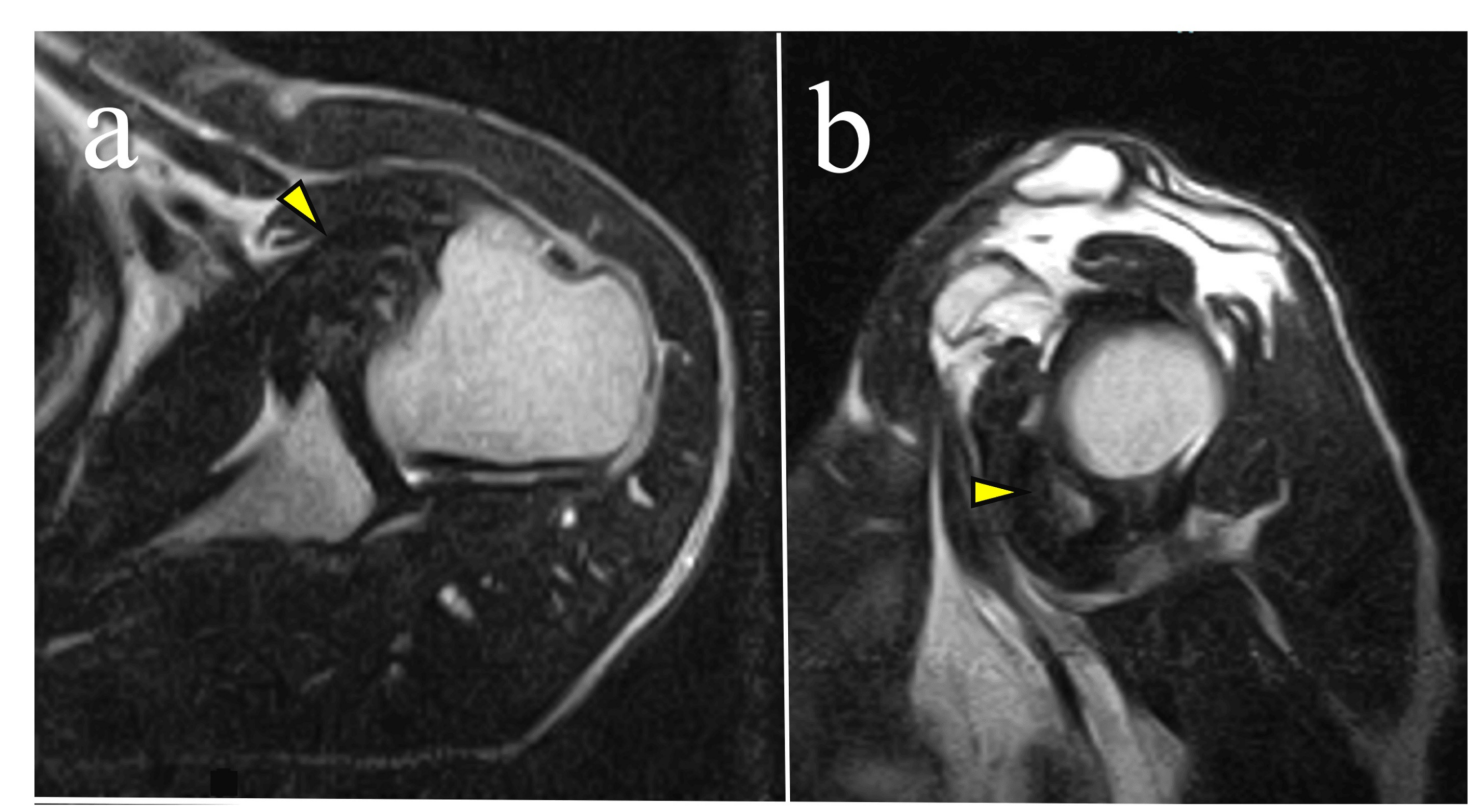
Magnetic resonance imaging obtained six years before referral to our institution. (a) Axial T1-weighted and (b) sagittal T2-weighted images demonstrating a space-occupying lesion deep to the subscapularis muscle (arrowheads) with low signal intensity on both sequences.

**Figure 3:**
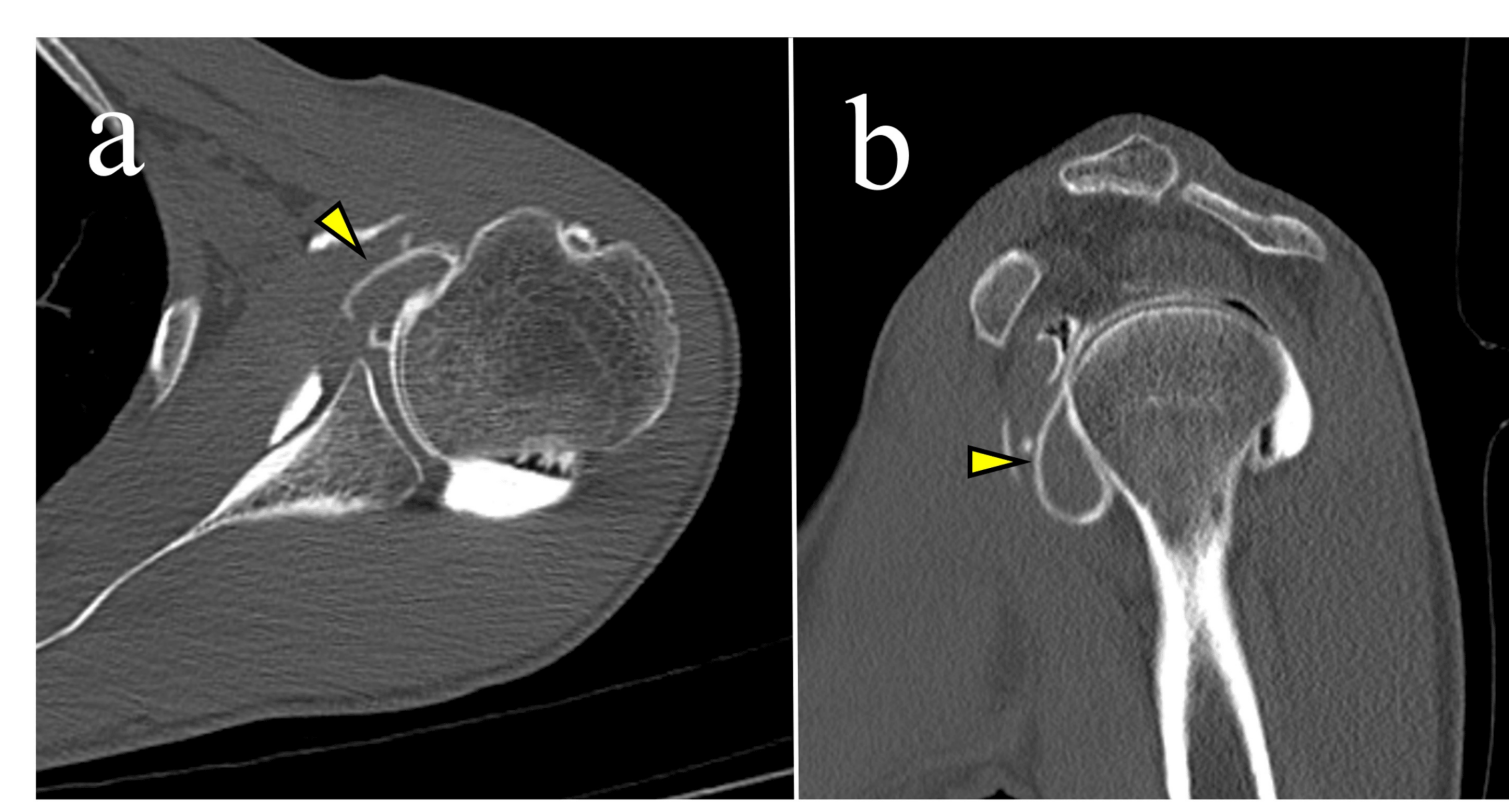
Intra-articular contrast-enhanced computed tomography obtained six years before referral to our institution (a) Axial and (b) sagittal images demonstrating an anterior intra-articular contrast filling defect (arrowheads) with peripheral contrast enhancement, suggestive of an intra-articular mass.

A 62-year-old man presented to a referring hospital with left shoulder pain at rest and during motion. He had no significant medical history or identifiable precipitating event. Plain radiographs of the shoulder showed no apparent abnormal findings (Figure 1). Magnetic resonance imaging (MRI) and intra-articular contrast-enhanced computed tomography (CT) subsequently revealed an intra-articular mass measuring 30 × 28 × 10 mm; however, the lesion was overlooked at the initial evaluation (Figures 2, 3). The shoulder pain did not improve over the following six years, during which the patient underwent conservative management, including observation, physical therapy, and evaluation for possible cervical spine pathology.

**Figure 4:**
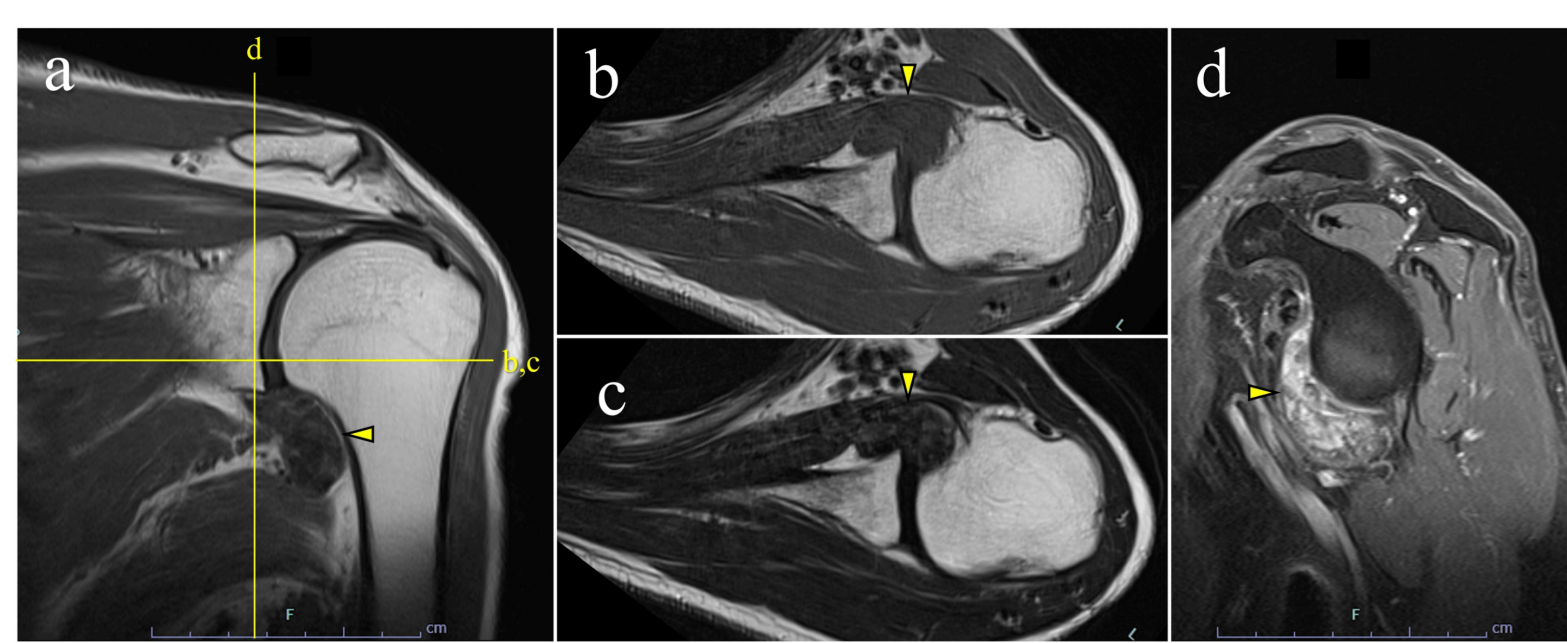
Magnetic resonance imaging findings at presentation. (a) Coronal proton density–weighted image demonstrating an intra-articular mass in the axillary pouch (arrowhead). No apparent rotator cuff tear was observed. (b) Axial T1-weighted image and (c) axial T2-weighted image showing a well-defined intra-articular mass with low signal intensity on both sequences, suggestive of a fibrous tumor (arrowheads). (d) Axial contrast-enhanced fat-suppressed T1-weighted image demonstrating heterogeneous internal enhancement of the lesion (arrowhead).

At the age of 68 years, he was referred to our institution for further evaluation and treatment of persistent shoulder pain. Active range of motion (ROM) of the left shoulder was restricted compared with the contralateral side (values are shown as right/ left): forward elevation was 160°/ 100°, external rotation at the side was 5°/ 0°, and external rotation at 90° of abduction was 40°/ 20°. Internal rotation behind the back reached the T9 level on the right and the L5 level on the left. The patient experienced pain during shoulder motion, resulting in limitations in activities of daily living that required elevation, reaching, and hand-behind-the-back movements. He also reported persistent resting pain with frequent nocturnal awakenings. The visual analog scale (VAS) score for resting pain was 100 mm (0 = no pain, 100 = worst imaginable pain) [7]. The American Shoulder and Elbow Surgeons (ASES) score was 37, with scores ranging from 0 to 100 and higher scores indicating better shoulder function [8,9]. MRI demonstrated an intra-articular mass located in the anteroinferior aspect of the shoulder joint, showing low signal intensity on both T1- and T2-weighted images with heterogeneous contrast enhancement, suggestive of a fibrous tumor (Figure 4). The tumor had well-defined margins and had increased in size to 30 × 48 × 23 mm compared with imaging obtained six years earlier.

**Figure 5:**
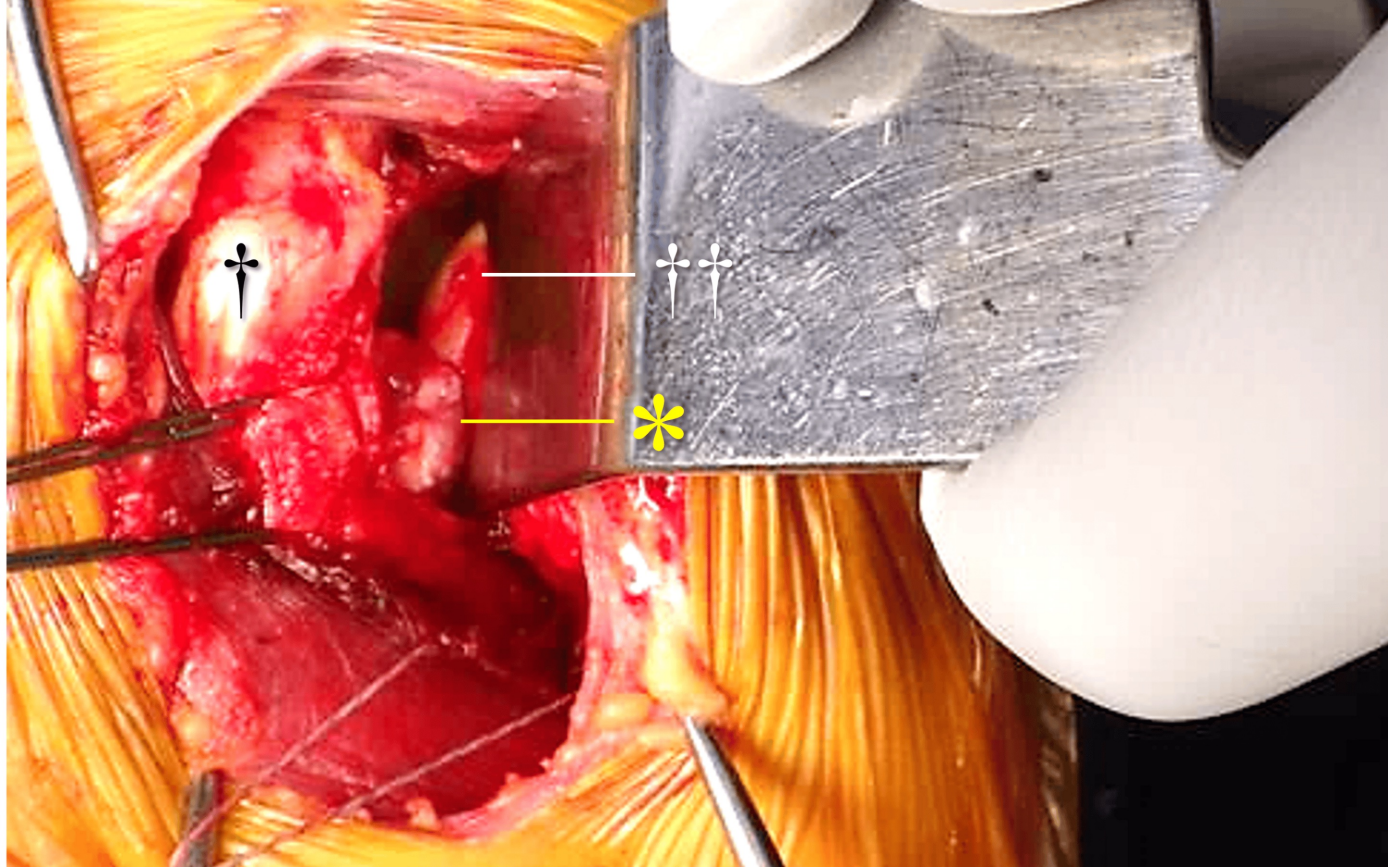
Intraoperative findings. After longitudinal splitting and reflection of the subscapularis muscle (†), a ring retractor was placed posterior to the glenoid to retract the humeral head (††) laterally. A well-circumscribed, pedunculated tumor (*) was identified arising from the anteroinferior joint capsule.

**Figure 6:**
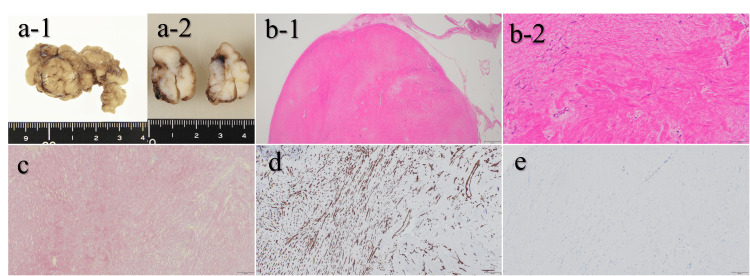
Histopathological findings of the resected tumor. (a) Gross appearance (a-1) and cut surface (a-2) of the tumor. The mass was lobulated, well circumscribed, and white to gray in color, with a homogeneous fibrous appearance and no evidence of hemorrhage or necrosis. (b) Hematoxylin and eosin staining at low (b-1, ×20) and high magnification (b-2, ×200). The tumor is well demarcated and composed of densely packed collagen bundles with sparsely distributed spindle- to stellate-shaped fibroblastic cells. No nuclear atypia, mitotic activity, or necrosis is observed. (c) Elastica van Gieson staining (×100) showing no significant increase in elastic fibers, supporting that the tumor is predominantly composed of collagen fibers. (d) Immunohistochemical staining for vimentin (×100) demonstrating diffuse cytoplasmic positivity in tumor cells, consistent with a mesenchymal (fibroblastic) origin. (e) Immunohistochemical staining for smooth muscle actin (×100) showing negative results, indicating no evidence of myofibroblastic or smooth muscle differentiation. Based on these findings, the tumor was diagnosed as collagen fibroma.

We performed surgical excision of a painful intra-articular tumor via a deltopectoral approach (Figure 5). Given the relatively large tumor size, we selected open excision rather than an arthroscopic approach to ensure complete removal under direct visualization. The subscapularis tendon and anterior joint capsule were incised longitudinally with a margin of approximately 2 cm from their insertion at the lesser tuberosity. A ring retractor was placed posterior to the glenoid to obtain adequate intra-articular visualization. After reflecting the subscapularis muscle, an intra-articular tumor was identified. The tumor was well circumscribed and pedunculated, with a stalk continuous with the anteroinferior joint capsule. The stalk was resected at its base along the capsule, and the tumor was completely excised. The subscapularis tendon was repaired with an end-to-end suture using a high-strength braided polyethylene suture. Histopathological examination confirmed the diagnosis of collagen fibroma (Figure 6).

Postoperative course

**Figure 7:**
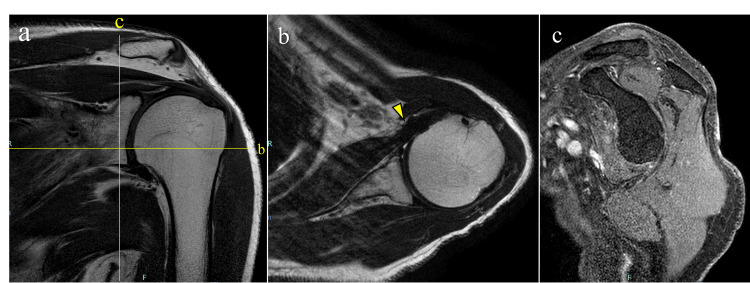
Magnetic resonance imaging at one year postoperatively. (a) Coronal T2-weighted image demonstrating no evidence of residual or recurrent tumor in the axillary pouch. (b) Axial T2-weighted image showing no tumor in the deep layer of the subscapularis muscle; the repaired subscapularis muscle is continuous (arrowhead). (c) Sagittal contrast-enhanced fat-suppressed T1-weighted image demonstrating no abnormal contrast enhancement suggestive of tumor recurrence.

After surgery, the patient was placed in a sling for two weeks and then began range-of-motion exercises as tolerated. At one month after surgery, resting pain and nocturnal pain had resolved rapidly. At one year postoperatively, the patient reported no left shoulder pain, and range of motion had improved to forward flexion of 160°/150°, external rotation at the side of 0°/0°, external rotation at 90° of abduction of 60°/60°, and internal rotation behind the back to the T9 level on the right and T10 level on the left. Follow-up MRI demonstrated no evidence of residual tumor or recurrence (Figure 7). The patient remained free of adverse events during five years of follow-up, and the ASES score at final follow-up was 100. A summary of the clinical course is provided in Table 1.

**Table 1:** Timeline of the clinical course

Age	Key events/findings
62	Onset of left shoulder pain; initial imaging performed, with the intra-articular mass not recognized at that time.
68	Referred to our institution; repeat imaging demonstrated tumor enlargement; open excision performed; the tumor was diagnosed as collagen fibroma.
73	Follow-up showed complete pain relief and no recurrence at 5 years postoperatively.

## Discussion

In the present case, collagen fibroma demonstrated an atypical clinical presentation in two respects: it was associated with pain and arose within the joint. To our knowledge, only two cases of intra-articular collagen fibroma have been reported to date [10,11], whereas most lesions occur in the subcutaneous or subfascial tissues [5]. Because collagen fibroma is typically painless and does not cause apparent clinical symptoms, many cases remain unrecognized and are left untreated for long periods [5]. In contrast, subcutaneous collagen fibroma may cause pain when it arises in regions with high soft-tissue tension and limited mobility, including the scalp or the plantar surface of the foot [12,13]. In our case, the tumor was located between the subscapularis muscle and the osseous structures of the scapula and humerus; thus, tumor enlargement likely increased tension on the anterior joint capsule and contributed to pain. Intra-articular tissues, particularly the joint capsule, ligaments, and synovium, are richly innervated with free nerve endings and nociceptors [14], and stretching or compression of these structures is a well-recognized mechanism of joint pain. Internal and external rotation of the shoulder alters the tension of the subscapularis muscle and the joint capsule, which may have further increased capsular stretching and provoked pain. The rapid resolution of both resting and motion-related pain after tumor excision strongly suggests that the symptoms were caused by localized mechanical irritation from the tumor.

The most important clinical issue was a diagnostic delay of six years. Collagen fibroma typically shows low signal intensity on both T1- and T2-weighted MRI [6], resulting in signal intensity similar to that of adjacent structures such as the subscapularis muscle and joint capsule. Consequently, the lesion provided little contrast against adjacent normal structures and was prone to being overlooked on routine shoulder MRI. When interpreting shoulder MRI, clinicians should keep fibrous tumors in mind and systematically evaluate the axillary pouch and subscapular recess on axial images, paying particular attention to subtle distortion of the anterior capsular contour, even when the lesion is isointense to surrounding tissues.

In this context, intra-articular lesions showing low signal intensity on MRI should be differentiated from other entities, including tenosynovial giant cell tumor (TGCT) and desmoid-type fibromatosis [4,6,15]. Unlike collagen fibroma, TGCT and desmoid-type fibromatosis frequently recur [16,17], making preoperative differentiation important for surgical planning. The diffuse type of TGCT, historically referred to as pigmented villonodular synovitis, shows hemosiderin-related low-signal intensity, often accompanied by joint effusion and bone erosion [16]. In contrast, desmoid-type fibromatosis shows strong contrast enhancement with internal fibrous low-signal bands and ill-defined margins due to its infiltrative growth pattern [11,17]. In evaluating low-signal intra-articular lesions, multiple imaging features should be interpreted in combination, including the degree of contrast enhancement, internal tumor characteristics (including hemosiderin deposition or fibrous bands), and the clarity of tumor margins. Therefore, intra-articular tumors should be included in the differential diagnosis of unexplained shoulder pain, and imaging findings should be interpreted in an integrated manner.

## Conclusions

This case represents a rare collagen fibroma arising within the shoulder joint and associated with severe pain. The tumor showed low signal intensity on both T1- and T2-weighted MRI and was therefore difficult to distinguish from surrounding structures, contributing to delayed diagnosis. Rapid resolution of pain after tumor excision suggested that the symptoms were caused by localized mechanical irritation from the tumor. In patients presenting with unexplained shoulder pain, careful imaging evaluation with consideration of intra-articular tumors, including fibrous tumors, is essential.
